# Investigating the burden of antibiotic resistance in ethnic minority groups in high-income countries: protocol for a systematic review and meta-analysis

**DOI:** 10.1186/s13643-017-0654-9

**Published:** 2017-12-11

**Authors:** Hannah Lishman, Paul Aylin, Vivian Alividza, Enrique Castro-Sanchez, Anuja Chatterjee, Victor Mariano, Alan P. Johnson, Samir Jeraj, Céire Costelloe

**Affiliations:** 1 NIHR Health Protection Research Unit in Healthcare-Associated Infections and Antimicrobial Resistance, Imperial College London, W12 0NN, London, UK; 2 Department of Healthcare-Associated Infections and Antimicrobial Resistance, National Infection Service, Public Health England, London, NW9 5EQ UK; 3 Race Equality Foundation, London, NW5 1LB UK

**Keywords:** Antibiotic resistance, Antibiotic prescribing, Ethnicity, High-income countries, Community-acquired infections, Healthcare-associated infections, Systematic review, Meta-analysis

## Abstract

**Background:**

Antibiotic resistance (ABR) is an urgent problem globally, with overuse and misuse of antibiotics being one of the main drivers of antibiotic-resistant infections. There is increasing evidence that the burden of community-acquired infections such as urinary tract infections and bloodstream infections (both susceptible and resistant) may differ by ethnicity, although the reasons behind this relationship are not well defined. It has been demonstrated that socioeconomic status and ethnicity are often highly correlated with each other; however, it is not yet known whether accounting for deprivation completely explains any discrepancy seen in infection risk. There have currently been no systematic reviews summarising the evidence for the relationship between ethnicity and antibiotic resistance or prescribing.

**Methods:**

This protocol will outline how we will conduct this systematic literature review and meta-analysis investigating whether there is an association between patient ethnicity and (1) risk of antibiotic-resistant infections or (2) levels of antibiotic prescribing in high-income countries. We will search PubMed/MEDLINE, EMBASE, Global Health, Scopus and CINAHL using MESH terms where applicable. Two reviewers will conduct title/abstract screening, data extraction and quality assessment independently. The Critical Appraisal Skills Programme (CASP) checklist will be used for cohort and case-control studies, and the Cochrane collaboration’s risk of bias tool will be used for randomised control trials, if they are included. Meta-analyses will be performed by calculating the minority ethnic group to majority ethnic group odds ratios or risk ratios for each study and presenting an overall pooled odds ratio for the two outcomes. The Grading of Recommendations, Assessments, Development and Evaluation (GRADE) approach will be used to assess the overall quality of the body of evidence.

**Discussion:**

In this systematic review and meta-analysis, we will aim to collate the available evidence of whether there is a difference in rates of AMR and/or antibiotic prescribing in minority vs. majority ethnic groups in high-income countries. Additionally, this review will highlight areas where more research needs to be conducted and may provide insight into what may cause differences in this relationship, should they be seen.

**Systematic review registration:**

PROSPERO (CRD42016051533)

**Electronic supplementary material:**

The online version of this article (10.1186/s13643-017-0654-9) contains supplementary material, which is available to authorized users.

## Background

Antibiotic resistance (ABR) is a serious threat to the treatment of infections in primary and secondary care in the UK and around the world. Optimising the use of antibiotics is a high priority as one of the main drivers of AMR is the misuse and overuse of antibiotics [1, 2]. This is a particular concern in high-income countries with ready access to antibiotics, as opposed to poorer countries where treatment of infections may be problematic due to the limited availability of these drugs [3].

There is increasing evidence that the burden of community-acquired infectious diseases such as urinary tract and bloodstream infections (both drug-susceptible and drug-resistant) or colonisation with multidrug-resistant (MDR) strains may differ by ethnicity [4–6], although the reasons behind this relationship are not yet clear. It has long been established that the concept of ethnicity in relation to disease burden is not one of biological difference as there is more genetic diversity within ethnic groups than between them, rather it is more relevant as a social construct [7]. It has been demonstrated in many studies that socioeconomic status and ethnicity are typically highly correlated with each other [4, 8, 9]; however, it is not yet known whether accounting for deprivation completely explains any discrepancy seen in infection risk between different communities. Could there be other factors at play?

It is therefore important to understand firstly whether the risk of AMR infections is disproportionately higher in minority or majority ethnic groups in settings where excess rather than access to antibiotics is more likely to be a problem. Secondly, if a difference is shown to exist, it will be key to understand why this difference is occurring and how it can be addressed.

There is increasing evidence that recent travel, particularly to South Asia (Bangladesh, India, Pakistan, Nepal, Sri Lanka etc.) and East Asia (China, Japan, Mongolia, Taiwan etc.), is a major risk factor for colonisation with drug-resistant bacteria leading to the risk of poorer health outcomes, treatment failure and subsequent transmission of these bacteria upon return to the country of residence [10, 11]. Several studies have confirmed that travel to certain geographical areas is an important risk factor for colonisation with extended spectrum beta-lactamase (ESBL) *Enterobacteriaceae*—with travel to the Indian subcontinent, Asia and Africa (north of the equator)—being areas of highest risk [12–16]. Among travellers, those particularly at risk of being colonised with multidrug-resistant bacteria are those visiting friends and family (indicating frequent travel to a particular region) and recent migrants [16].

Another possible factor which may account for differences in ABR burden may be that the level of awareness around appropriate antibiotic use varies with differing population attributes. In a study by McKee et al., 192 participants from an ethnically diverse urban community in the USA were interviewed about their health-seeking behaviours, their use of antibiotics without a prescription and their perception of the effectiveness of different treatments for upper respiratory tract infections (URTIs) [17]. The majority of participants believed that antibiotics were effective against URTIs, with 26% having obtained antibiotics either directly from a pharmacist or from a source outside the USA and 31% believed that antibiotics should be available over the counter. Worryingly, participants originally from countries where antibiotics were available over the counter were significantly more likely to use antibiotics without a prescription than those who were born in the USA or those born in countries where antibiotics were not available over the counter (*p* = 0.049) [17].

It is widely recognised that the knowledge, behaviour and attitudes of both the prescriber and the patient influence antibiotic prescribing [18]. It is therefore essential that public knowledge and behaviour change campaigns around the use of antibiotics reach all citizens. In a UK-based study by McNulty et al. [19], a questionnaire was included in the Office of National Statistics (ONS) Omnibus Household Survey in Britain to ascertain the public’s knowledge about antibiotics. Of the 7120 participants from England, Scotland and Wales, 38% did not know that antibiotics do not work on most coughs and colds, 4.8% had used an antibiotic without advice from a healthcare provider, 1.7% had given antibiotics to someone for whom they were not prescribed and 4.7% had obtained an antibiotic in another country without a prescription. When ethnicity was taken into consideration, participants of Asian or Black Caribbean ethnicity gave more responses indicating incorrect knowledge or use of antibiotics than White British participants [19].

There have been no systematic reviews or meta-analyses summarising the evidence for the relationship between ethnicity and antibiotic resistance or prescribing; this review is therefore warranted in order to collate the available evidence. The aims for the systematic review will be to determine if there is evidence for an association between patient ethnicity and (1) consumption of antibiotics or (2) risk of antibiotic-resistant infections in high-income countries. The overall aim of this paper is to outline a transparent method for screening, collecting and collating the data available along with describing the statistical methodology that will be used for a meta-analysis should the data allow for such an analysis to be conducted.

## Methods/design

To improve the quality of reporting, this protocol has been written in accordance with the Preferred Reporting Items for Systematic Reviews and Meta-Analyses for Protocols (PRISMA-P) guidelines (checklist can be found in Additional file 1). The protocol is registered with PROSPERO (CRD42016051533).

### Research questions

The following research questions were developed:

1. What evidence is there for an association between patient ethnicity and risk of antibiotic-resistant infections in high-income countries?
2. What evidence is there for an association between patient ethnicity and prescribing of antibiotics in high-income countries?

### Population

All men and women of any age living in any of the 35 high-income countries will be included. A high-income country will be defined as being a member country of the Organisation for Economic Co-operation and Development (OECD) high-income status group [20]. A list of the 35 OECD-defined high-income countries can be found in Additional file 2.

### Exposure

The exposure of interest will be patient ethnicity and will be defined by ethnicity reported in the health record or self-reported by the patient. We are interested in measuring the difference in rates of antibiotic resistance or antibiotic prescribing between minority and majority ethnic groups. The definition of “minority” and “majority” ethnic groups will differ depending on the country in which the study takes place and will be guided by how the ethnic groups under study are reported in the articles and verified by national census data where they are available.

### Comparator/control

The comparator will be patients falling into the “majority” ethnic group in the high-income country being studied. For example, in the UK, the majority ethnic group would be “White” whereas the minority ethnic groups would include all “Black” ethnic backgrounds (Black or Black British: Caribbean, African or Other Black background), all “Asian” ethnic backgrounds (Asian or Asian British: Indian, Pakistani, Bangladeshi, Chinese or other Asian background), all “Mixed or Multiple” ethnic backgrounds and “Other” ethnic backgrounds [21]. In Japan, however, the majority ethnic group would be “Japanese” and the minority ethnic groups would be “Korean”, “Chinese” and “American” [22].

### Outcomes

The primary outcomes of the included studies will be either infection-related outcomes or antibiotic prescribing-related outcomes as outlined below. The reporting of either of these outcomes will be mandatory for inclusion.

1. Infection-related outcomes

-Prevalence or incidence of antibiotic-resistant infections defined as the number of new or existing antibiotic-resistant infections per 1000 population (for community rates) or per 1000 admissions or occupied bed days (OBD) (for hospital rates).
-Percentage (%) of infections resistant to an antibiotic class which should otherwise be effective against that pathogen.

2. Antibiotic prescribing-related outcomes

-Prescribing or consumption of antibiotics measured as defined daily doses (DDD) or number of items per 1000 population (for community rates) or DDD or number of items per 1000 admissions or OBD (for hospital rates).

Secondary outcomes of the included studies will be the study setting (prescribing in primary care/community-acquired infections vs. prescribing in secondary care/hospital-acquired infections) and age (children vs. adults). These outcomes will not be mandatory for inclusion but it is expected that they will be reported in most included studies.

### Inclusion/exclusion criteria

Observational studies including cohort (retrospective and prospective), case-control, cross-sectional, longitudinal and ecological study designs conducted in high-income countries and containing quantitative data on antibiotic-resistant infection/colonisation or antibiotic prescribing by ethnic group will be included. Interventional studies (randomised controlled trials) will be included if they meet the criteria in Part A; however, we do not expect that there have to be such studies in this area. Only articles written in or translated into English will be included. All age groups, genders and time periods will be included.

Studies will not be included if they are set in middle- or low-income countries, defined as countries which are not members of the OECD high-income status group. All studies focussing exclusively on tuberculosis, HIV/AIDS, malaria, viral hepatitis or sexually transmitted infections such as chlamydia, syphilis or gonorrhoea will be removed during screening as these infections have been widely studied separately and are beyond the scope of this review. Clinical case studies, case reports and systematic reviews or meta-analyses will also be excluded during screening. Studies set in intensive care units only or studies using entire countries as comparators will be excluded. Finally, studies written in a language other than English will be excluded.

### Search strategy

The search strategy will include electronic database searching, citation searching of the included full-text articles and a grey literature search. The following databases will be searched: MEDLINE via PubMed, EMBASE, Global Health, Scopus and CINAHL. Reference lists of the included articles will be screened for relevant articles not previously found in the database searches. Grey literature will be reviewed using Google Scholar and OpenGrey. Dissertations and theses will be searched for in ProQuest Dissertations and Theses. Search terms will be tailored for each database, using MeSH terms where applicable, and will be checked by a medical librarian to ensure the syntax is correct.

No study design or date limits will be imposed on the search, although only studies in English will be included due to limited resources. Medline, EMBASE and Global Health databases will be searched through Ovid; Scopus and CINAHL databases will be searched separately. The specific search strategies will be created with the assistance of a Health Sciences Librarian with expertise in systematic review searching. The search terms are based around the concepts of “ethnicity”, “antibiotic prescribing”, “antibiotic resistance” and “high-income countries”. The “ethnicity”-related search terms have been informed by a Cochrane review investigating culturally appropriate health education for type 2 diabetes in minority ethnic groups [23]. The PubMed/MEDLINE search strategy is included in Additional file 3 as an example. These search terms will then be adapted to the syntax and subject headings of the other databases. The search will be updated toward the end of the review to ensure that all information in the review is up-to-date. The publications from the five databases will be exported into EPPI Reviewer software (https://eppi.ioe.ac.uk/cms/Default.aspx?tabid=2914), where duplicates will be removed.

All titles and abstracts yielded by the search will be independently screened by two authors (HL for all articles and ECS, AC, VA or VM as second reviewers) to ensure consistency of the application of the inclusion/exclusion criteria. Full texts will be obtained for all titles that appear to meet the inclusion criteria and any discrepancies will be resolved by discussion among the review team or by the principal investigator. Additional information will be sought from study authors where necessary to resolve questions about eligibility. Reasons for excluding studies will be recorded. Once full texts have been obtained, all reviewers will perform data extraction, HL on the entire set of included studies and ECS, AC, VA and VM on a quarter of the studies each.

Reference lists of the included studies will be searched for relevant articles which were missed by the original database search. Relevant title/abstracts will be screened against the inclusion and exclusion criteria and will be reviewed independently by two authors in the same way as for the database search. Full texts will be retrieved from included articles and data will be extracted.

Grey literature will be reviewed by searching for relevant documents in OpenGrey, Google Scholar and ProQuest Dissertations and Theses. In OpenGrey, the following search will be performed: ((antibiotic) OR (antimicrobial)) AND ((ethnic*) OR (socio*)). In Google Scholar, the following search will be performed: “antibiotic” AND “ethnic*” and the first 100 articles will be reviewed. In ProQuest Dissertations and Theses, the following search will be performed: ab,ti(antibiotic*) AND ab,ti(ethnic*). The documents will be screened against the inclusion and exclusion criteria and reviewed independently by two authors in the same way as for the database search.

### Quality assessment

The reviewers will independently assess the quality of the included full-text articles. The quality and risk of bias of observational studies will be assessed using the Critical Appraisal Skills Programme (CASP) checklist for cohort and case-control studies independently by the review team and discussed if discrepancies occur (www.casp-uk.net). Cross-sectional studies will be quality assessed using the Appraisal tool for Cross-Sectional Studies (AXIS tool) [24]. If randomised control trials are included, the Cochrane collaboration’s risk of bias tool will be used to assess quality. A quality assessment chart based on a traffic light system of “good”, “adequate” and “poor” reporting will be developed as recommended by Cochrane [25].

### Data analysis and synthesis

Data extraction will be performed independently by two reviewers using a customised form in EPPI Reviewer. Data extracted will include first author, year of publication, year(s) of study, country, aim/hypothesis, study design, number of participants (N), age range of participants, setting (community or hospital), urban or rural area, individual or population level exposure, ethnic categories for comparison, which groups are exposure group and which are control (minority vs. majority ethnic group in that country), outcome measured (antibiotic prescribing or antibiotic resistance or both), how outcome was measured (defined daily doses (DDD)/1000 population, DDD/1000 admissions, DDD/1000 OBD, incidence or prevalence of resistant infection, % resistance in a group etc.), unadjusted relative difference between groups (risk ratio, odds ratio etc.), variables adjusted for (such as age, sex, comorbidities, lifestyle factors), and adjusted relative difference between groups.

The data synthesis will depend upon the level of heterogeneity of the extracted data. Heterogeneity will be assessed by generating forest plots of the individual included studies to examine confidence intervals, using the *χ* ^2^ test to determine whether there is evidence of significant heterogeneity between the studies (*p* < 0.10) and calculating the *I* ^2^ statistic to estimate the level of heterogeneity using values of 30 to 60%, 50 to 90% and 75 to 100% to indicate moderate, substantial and considerable heterogeneity, respectively [25]. Based on the outcomes of these tests combined, if a quantitative analysis is not possible due to the data being too heterogeneous, a narrative synthesis will be provided to summarise the results displayed in the included studies. This will include providing tables and summary measures based on the primary outcomes (prescribing and resistance rates) stratified by the secondary outcomes (hospital vs. community settings, children vs. adults). Due to the broad scope of this systematic literature review, heterogeneity is expected with respect to study design, study populations and the reporting of exposures (ethnicity) and outcomes (prescribing or resistance). Separate tables will be created for each of the two primary outcomes to explore these factors and compare and contrast the results of the studies within and between the subgroups (hospital vs community and children vs adult).

-Community setting will be comprised of either antibiotic prescribing at the general practitioner (GP) practice (DDD or number of items per 1000 population) or incidence of antibiotic-resistant infections presenting at the GP practice or within two days of admission to hospital (community-associated infections).
-Hospital setting will be comprised of either antibiotic prescribing for an admitted patient in hospital (DDD or number of items per 1000 OBD or admissions) or incidence of antibiotic-resistant infections presenting at least two days after admission to hospital (hospital-acquired infections).
-Adults will be patients 18 years of age or older; children will be patients 0–17 years of age.

Where homogenous data allow, meta-analyses will be performed using random effect models (based on the variables specified). Where outcomes are continuous, they will be transformed to categorical or binary variables as appropriate. The minority ethnic group to majority ethnic group odds ratios for each included study will then be calculated in a similar way to the Ethnic Minority Meta-Analysis (EMMA) [26], and overall pooled odds ratios with 95% confidence intervals for the two outcomes (and/or sub analyses) will be presented from the random effects models. Should more than one randomised controlled trial be included, separate meta-analyses will be performed for the trials and for the included observational studies. All analyses will be performed in EPPI Reviewer.

### Meta-bias(es) and confidence in cumulative evidence

If the number of included articles is high enough and if the data allow, funnel plots will be used to detect the presence of publication bias. The Grading of Recommendations, Assessments, Development and Evaluation (GRADE) approach will be used to assess the quality of the body of evidence and a summary of findings table will be used to present the results.

### Dissemination

The systematic review and meta-analysis will be submitted for publication in a relevant journal. The results will be presented at conferences that are in line with the topic area. This work will contribute to a PhD project as part of the National Institute for Health Research Health Protection Research Unit (NIHR HPRU) in Healthcare-Associated Infections and Antimicrobial resistance at Imperial College London. The results of the review will be communicated to public and patient populations through the Race Equality Foundation dissemination networks as well as through public announcements by the HPRU.

## Discussion

In this systematic review and meta-analysis, we will aim to collate and present the available evidence of whether there is a difference in rates of AMR and/or antibiotic prescribing by minority vs. majority ethnic groups in high-income countries. In this way, this review will contribute to a wider body of knowledge of the role of socio-demographic factors such as poverty and minority ethnic background may play in the risk of antibiotic-resistant infections in resource-rich countries where excess of antibiotics is of greater concern than access. The results of this review will describe this relationship and highlight areas where more research needs to be conducted and may provide insight into possible reasons for any differential levels of risk that may be seen.

Some possible limitations of this review that can be anticipated at this stage are (i) the English language restriction on the included articles due to resource limitations in the review team and (ii) that there are not likely to be any articles from France as they do not record ethnicity in the national census. These potential limitations will be taken into consideration when interpreting the findings of this review.

## Additional files

Additional file 1: PRISMA-P checklist. The Preferred Reporting Items for Systematic Reviews and Meta-Analyses for Protocols (PRISMA-P) checklist was used to develop this protocol. (DOCX 29 kb)

Additional file 2: List of OECD high-income countries. List of countries classified as “high income” by the Organisation for Economic Co-operation and Development (OECD). (DOCX 12 kb)

Additional file 3: Search terms for MEDLINE. The search terms that will be used to identify relevant literature in MEDLINE as an example, adapted for the other four databases. (DOCX 14 kb)

## Abbreviations

ABR: Antibiotic resistance
CASP: Critical Appraisal Skills Programme
DDD: Defined daily dose
ESBL: Extended spectrum beta-lactamase
GRADE: The Grading of Recommendations, Assessments, Development and Evaluation
MDR: Multidrug resistant
NIHR HPRU: National Institute for Health Research Health Protection Research Unit
OBD: Occupied bed day
URTI: Upper respiratory tract infection
