# Supplementary material for: Investigating the burden of antibiotic resistance in ethnic minority groups in high-income countries: protocol for a systematic review and meta-analysis
Source: Syst Rev. 2017 Dec 11;6:251. doi: 10.1186/s13643-017-0654-9 (PMC5725910; doi:10.1186/s13643-017-0654-9)
Supplement: Supplementary file 2 — List of OECD high-income countries. List of countries classified as “high income” by the Organisation for Economic Co-operation and Development (OECD). (DOCX 12 kb) [file 13643_2017_654_MOESM2_ESM.docx]

**OECD high-income countries:**

1. Australia

2. Austria

3. Belgium

4. Canada

5. Chile

6. Czech Republic

7. Denmark

8. Estonia

9. Finland

10. France

11. Germany

12. Greece

13. Hungary

14. Iceland

15. Ireland

16. Israel

17. Italy

18. Japan

19. Korea

20. Latvia

21. Luxembourg

22. Mexico

23. Netherlands

24. New Zealand

25. Norway

26. Poland

27. Portugal

28. Slovak Republic

29. Slovenia

30. Spain

31. Sweden

32. Switzerland

33. Turkey

34. United Kingdom

35. United States
