# Supplementary material for: Investigating the burden of antibiotic resistance in ethnic minority groups in high-income countries: protocol for a systematic review and meta-analysis
Source: Syst Rev. 2017 Dec 11;6:251. doi: 10.1186/s13643-017-0654-9 (PMC5725910; doi:10.1186/s13643-017-0654-9)
Supplement: Supplementary file 3 — Search terms for MEDLINE. The search terms that will be used to identify relevant literature in MEDLINE as an example, adapted for the other four databases. (DOCX 14 kb) [file 13643_2017_654_MOESM3_ESM.docx]

# Systematic review terms

**MEDLINE (Ovid)**

1. Exp Ethnic Groups/

2. Exp Continental Population Groups/

3. ethnic*.tw,ot

4. race.mp

5. racial.mp

6. caucasian.mp

7. asian.mp

8. african.mp

9. hispanic.mp

10. exp Minority Groups/
11. exp Multilingualism/
12. exp Refugees/
13. exp Population Groups/
14. exp Hispanic Americans/
15. exp African Continental Ancestry Group/
16. exp American Native Continental Ancestry Group/
17. exp Asian Continental Ancestry Group/
18. exp European Continental Ancestry Group/
19. exp Oceanic Ancestry Group/
20. exp African Americans/
21. exp Arabs/
22. exp Asian Americans/
23. exp Gypsies/
24. exp Mexican Americans/
25. exp Inuits/
26. exp Jews/
27. exp Indians, South American/ or exp Indians, North American/
28. exp Cultural Characteristics/
29. ((underserve* or disadvantage*) adj6 (group* or population*)).tw,ot.
30. (multi ethnic* or multi?ethnic*).tw,ot.
31. (multi?racial* or multi racial*).tw,ot.
32. (migrant* or immigrant*).tw,ot.
33. refugees.tw,ot.
34. asylum seeker*.tw,ot.
35. cultural diversit*.tw,ot.
36. (multi?lingual or multi lingual).tw,ot.
37. (multi?cultural or multi cultural or cross?cultural or cross cultural or trans?cultural or transcultural).tw,ot.
38. exp Islam/
39. exp Hinduism/
40. exp Buddhism/
41. (islam* or hindu* or sikh* or buddhism*).tw,ot.

42. 1 or 2 or 3 or 4 or 5 or 6 or 7 or 8 or 9 or 10 or 11 or 12 or 13 or 14 or 15 or 16 or 17 or 18 or 19 or 20 or 21 or 22 or 23 or 24 or 25 or 26 or 27 or 28 or 29 or 30 or 31 or 32 or 33 or 34 or 35 or 36 or 37 or 38 or 39 or 40 or 41

43. exp Developed Countries/

44. high?income adj3 countr*.ab,ti.

45. exp Australia/

46. exp Austria/

47. exp Belgium/

48. exp Canada/

49. exp Chile/

50. exp Czech Republic/

51. exp Denmark/

52. exp Estonia/

53. exp Finland/

54. exp France/

55. exp Germany/

56. exp Greece/

57. exp Hungary/

58. exp Iceland/

59. exp Ireland/

60. exp Israel/

61. exp Italy/

62. exp Japan/

63. exp Korea/

64. exp Latvia/

65. exp Luxembourg/

66. exp Mexico/

67. exp Netherlands/

68. exp New Zealand/

69. exp Norway/

70. exp Poland/

71. exp Portugal/

72. exp Slovak Republic/

73. exp Slovenia/

74. exp Spain/

75. exp Sweden/

76. exp Switzerland/

77. exp Turkey/

78. exp Great Britain/

79. exp United States/

80. developed countr$.ab,ti or australia.ab,ti or austria.ab,ti or belgium.ab,ti or canada.ab,ti or chile.ab,ti or czech republic.ab,ti or denmark.ab,ti or estonia.ab,ti or finland.ab,ti or france.ab,ti or germany.ab,ti or greece.ab,ti or hungary.ab,ti or iceland.ab,ti or ireland.ab,ti or israel.ab,ti or italy.ab,ti or japan.ab,ti or korea.ab,ti or latvia.ab,ti or luxembourg.ab,ti or mexico.ab,ti or netherlands.ab,ti or new zealand.ab,ti or norway.ab,ti or poland.ab,ti or portugal.ab,ti or slovak republic.ab,ti or slovenia.ab,ti or spain.ab,ti or sweden.ab,ti or switzerland.ab,ti or turkey.ab,ti or great britain.ab,ti or united kingdom.ab,ti or united states.ab,ti

81. 43 or 44 or 45 or 46 or 47 or 48 or 49 or 50 or 51 or 52 or 53 or 54 or 55 or 56 or 57 or 58 or 59 or 60 or 61 or 62 or 63 or 64 or 65 or 66 or 67 or 68 or 69 or 70 or 71 or 72 or 73 or 74 or 75 or 76 or 77 or 78 or 79 or 80

82. anti?bacterial agent$.mp

83. antibiotic$.mp

84. antimicrobial$.mp

85. drug resistan$.mp

86. microbial sensitivity test$.mp

87. exp Anti-Bacterial Agents/

88. exp Anti-Infective Agents/

89. exp Drug Resistance, Bacterial/

90. exp Drug Resistance, Multiple, Bacterial/

91. exp Microbial Sensitivity Tests/

92. 52 or 53 or 54 or 55 or 56 or 57 or 58 or 59 or 60 or 61

93. 12 and 51 and 62

94. Limit 63 to english language
